# Current Modulation of a Heterojunction Structure by an Ultra-Thin Graphene Base Electrode

**DOI:** 10.3390/ma11030345

**Published:** 2018-02-27

**Authors:** Carlos Alvarado Chavarin, Carsten Strobel, Julia Kitzmann, Antonio Di Bartolomeo, Mindaugas Lukosius, Matthias Albert, Johann Wolfgang Bartha, Christian Wenger

**Affiliations:** 1 IHP, Im Technologiepark 25, 15236 Frankfurt (Oder), Germany; kitzmann@ihp-microelectronics.com (J.K.); lukosius@ihp-microelectronics.com (M.L.); wenger@ihp-microelectronics.com (C.W.); 2 Technische Universität Dresden, Semiconductor and Microsystems Technology Laboratory, 01062 Dresden, Germany; carsten.strobel@tu-dresden.de (C.S.); matthias.albert@tu-dresden.de (M.A.); johann.bartha@tu-dresden.de (J.W.B.); 3 Physics Department E R Caianiello, University of Salerno, via Giovanni Paolo II, I-84084 Fisciano, Salerno, Italy; dibant@fisica.unisa.it; 4 Medical High School Theodor Fontane, 16816 Neuruppin, Germany

**Keywords:** graphene, amorphous silicon, vertical transistors

## Abstract

Graphene has been proposed as the current controlling element of vertical transport in heterojunction transistors, as it could potentially achieve high operation frequencies due to its metallic character and 2D nature. Simulations of graphene acting as a thermionic barrier between the transport of two semiconductor layers have shown cut-off frequencies larger than 1 THz. Furthermore, the use of n-doped amorphous silicon, (n)-a-Si:H, as the semiconductor for this approach could enable flexible electronics with high cutoff frequencies. In this work, we fabricated a vertical structure on a rigid substrate where graphene is embedded between two differently doped (n)-a-Si:H layers deposited by very high frequency (140 MHz) plasma-enhanced chemical vapor deposition. The operation of this heterojunction structure is investigated by the two diode-like interfaces by means of temperature dependent current-voltage characterization, followed by the electrical characterization in a three-terminal configuration. We demonstrate that the vertical current between the (n)-a-Si:H layers is successfully controlled by the ultra-thin graphene base voltage. While current saturation is yet to be achieved, a transconductance of ~230 μS was obtained, demonstrating a moderate modulation of the collector-emitter current by the ultra-thin graphene base voltage. These results show promising progress towards the application of graphene base heterojunction transistors.

## 1. Introduction

Electronic devices with vertical transport and architecture have gained attention as a new path into greater performances. Additionally, the advent of 2D materials and their physics could play a decisive role for operation levels not yet achieved by traditional semiconductors. Specifically, graphene has been proposed by Mehr et al. [1] in 2012 to be used as an ultra-thin base electrode to modulate the vertical transport barrier of heterojunction transistors. Simulations demonstrated attainable operation frequencies in the THz range. The proposed structure composed of dielectrics embedding graphene is based on the field emission of hot electrons as the charge transport mechanism. This was experimentally demonstrated by two different groups in 2013 [2,3]. To achieve the targeted operation frequencies, the insulators were simulated to be 2–3 nm thin with a low Schottky barrier (0.4 eV) at the metal contact [1]. However, in the experimental reports, 5 nm oxides with barriers of about 3 eV were used, thus showing low current values (<1 µA/cm^2^) and current gains (<0.1) [2,3]. To alleviate these band engineering requirements, Di Lecce et al. [4] proposed to replace the dielectrics in the vertical transistor by n-doped crystalline silicon (n-Si) to embed graphene. In this case, thermionic emission is expected to be the dominant current transport mechanism. Under the assumption that the graphene monolayer is undoped or p-doped, the so-called graphene-base heterojunction transistor (GBHT) can be seen as a variation of the n-p-n Bipolar Transistor [4].

Along with high operation frequencies, mechanical flexibility is another desired feature in the new generation of bendable electronic devices. In this field, graphene has positioned itself as a promising candidate [5]. Hydrogen-passivated amorphous silicon (a-Si:H) has also been successfully used for flexible electronics such as displays [6] and strain sensors [7]. Thus, by replacing the brittle crystalline n-Si from the original device concept with n-doped a-Si:H for embedding graphene, a flexible GBHT operating at high frequencies could be realized. Although graphene Schottky diodes with rigid silicon layers have been studied extensively, see e.g., the review of Di Bartolomeo [8], the interfaces of graphene with flexible layers such as a-Si:H have been scarcely investigated. The non-trivial task of growing a-Si:H layers on graphene has been studied in the past by means of electron-beam deposition [9] and plasma-enhanced chemical vapor deposition (PECVD) [10]. Lupina et al. [11] reported the use of very high plasma excitation frequencies (140 MHz) instead of the commonly used radio frequency (13.56 MHz) during PECVD, allowing the deposition of a-Si:H layers on graphene without inducing damage to the underlying sheet. This was attributed to a reduction of the ion energies in the plasma due to the increased excitation frequency [12]. The damage-free deposition of a-Si:H by Very High Frequency PECVD (VHF-PECVD) on graphene allowed the electrical characterization of the (n)-a-Si:H/graphene junction by Strobel et al. [13], which reported a Schottky barrier of 0.35–0.49 eV (depending on the substrate) and promising large rectification ratios of up to 10^5^. Here we present the electrical characterization of a graphene layer embedded between two (n)-a-Si:H layers deposited by VHF-PECVD and the ability of graphene to modulate the vertical current in the structure up to 40%.

This manuscript is organized as follows. In Section 2, the experimental methods for the deposition of a-Si:H and the transfer of graphene are explained. In Section 3, the results of the current-voltage analysis of the two graphene/silicon interfaces will be presented. Afterwards, the three-terminal operation of the vertical structure in a common emitter configuration will be shown and discussed in Section 3. Finally, the main results are summarized in Section 4.

## 2. Materials and Methods

**Figure 1:**
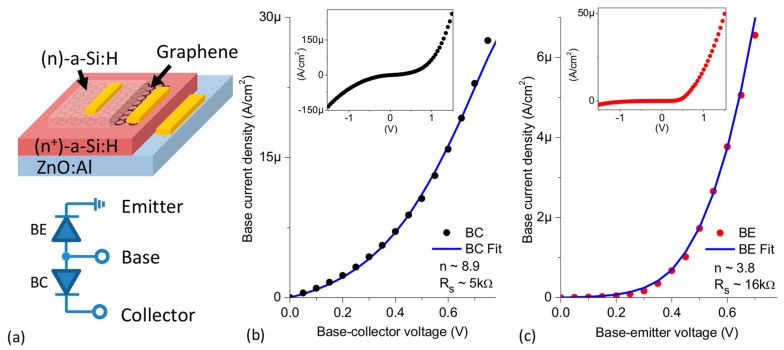
(**a**) Schematic illustration of a graphene monolayer (black) embedded by two (n)-a-Si:H layers (the top layer is the emitter and the bottom layer is the collector). Forward-bias output characteristics of the (**b**) BC (base-collector) and (**c**) BE (base-emitter) interfacial diodes. Insets: Forward and backward bias of the (**b**) BC and (**c**) BE diodes.

In Figure 1a, the simplified scheme of the embedded graphene in a GBHT configuration is shown. The collector layer consists of 100 nm (n^+^)-a-Si:H deposited over a ZnO:Al substrate by VHF-PECVD (140 MHz) at a constant temperature of 180 °C using a gas mixture of silane, hydrogen and 0.1% phosphine (PH_3_) as n-type dopant. The deposition system is described in detail elsewhere [14]. By increasing the doping gas ratio in the gas mixture, a thin layer (~10 nm) of highly doped amorphous silicon was deposited to ensure an ohmic contact with the metallic electrode. This was confirmed experimentally by a linear IV relation. Afterwards, a 1 cm^2^ single layer of CVD graphene grown on Cu foil (by Graphenea S.A.) was transferred onto the surface of the (n^+^)-a-Si:H (collector) layer by the polymer-assisted (PMMA) method. To remove any oxide from the surface of the (n^+^)-a-Si:H layer, the sample was dipped in 1% HF for 30 s. It was also observed that the HF treatment improved the quality and integrity of the graphene transfer. After the removal of the sacrificial PMMA layer with acetone, another 100 nm (n)-a-Si:H layer was deposited on the surface of graphene using a low power VHF-PECVD regime with 0.0225% PH_3_ as dopant. A section of the graphene layer was protected from the a-Si:H deposition to place the metal electrode. Due to restrictions in the fabrication process, the deposited emitter layer partially exceeds the surface of the graphene layer. The conductivities of the emitter and collector layers are 5 × 10^−5^ S/cm and 3 × 10^−3^ S/cm, respectively. Although heavily doped emitter is the usual configuration to achieve high gain, the highly doped collector layer was chosen since an improvement of the cut off frequency has been reported [15]. Aluminum contacts were thermally evaporated on the surface of the three layers using a shadow mask under high vacuum conditions. The electrical characterization was done using a Keithley 4200 system and a PMV chamber with Cascade micropositioners employing tungsten tips.

## 3. Results and Discussion

### 3.1. Diode Characterization

The base-collector (BC) and base-emitter (BE) IV characteristics were analyzed independently in the same structure by applying a voltage to the graphene contact while leaving the other contact grounded. Assuming an undoped or p-doped graphene channel, we expected a diode-like behavior between graphene and both (n)-a-Si:H layers. The assumption of a p-doped graphene layer is in agreement with reports of transferred graphene grown on Cu foil by CVD [16,17,18] as being the prevailing condition. The output characteristics in forward bias are illustrated in Figure 1b,c, while the insets show the forwards and backwards biased set-up of the BC and BE diodes, respectively. It can be seen that the BC diode (Figure 1b) exhibits a negligible rectifying behavior with a current rapidly increasing both in forward and reverse bias. In comparison, the BE interface (Figure 1c) has a more noticeable diode-like and rectifying behavior. The results of the BE diode can be explained considering the lower doped emitter substrate, which agrees with the observation that graphene/Si diodes demonstrated higher rectification on lightly doped substrates [8]. Likewise, the behavior of the BC interface can be understood as a diode with a reduced barrier caused by the larger force image on highly doped substrates which lowers the Schottky barrier and reduces the rectification ratio. By extrapolating the linear part of the IV curves to the voltage axis in forward bias, we extracted a threshold voltage $V_{F}$~0.33 V for the BC diode and $V_{F}$~0.45 V for the BE diode.

A non-ideal diode behavior with a dominating thermionic transport mechanism is expected for the (n)-a-Si:H/graphene diodes [13]. Hence, the forward IV characteristics are given by the Schottky model

(1) $$J\left(V,T\right)=J_{0}\left(T\right)\left[exp\left(\frac{q(V-JA_{d}R_{s}}{nkT}\right)-1\right],$$

where J is the current density, $A_{d}$ the diode area, $J_{0}$ is the saturation current, n the ideality factor, k the Boltzmann constant and $R_{s}$ are the series resistances. Equation (1) was used as a model to fit the experimental data in forward bias and to obtain a first approximation of n and $R_{s}$ for both diodes. The BC diode demonstrates a larger ideality factor (n= 8.9) compared to that of the BE diode (n= 3.8), while $R_{s}$ was larger at the base-emitter interface (~16 kΩ) than at the base-collector interface (~5 kΩ). The discrepancy of $R_{s}$ between both junctions can be affected by the bulk resistance of the corresponding (n)-a-Si:H layer. The BE ideality factor is comparable to values often obtained in literature for n-Si/Graphene diodes [19,20]. Large n values are symptomatic of defect rich interfaces or inadvertent thin oxide layers and points towards a non-pure thermionic conduction mechanism [21]. More than 30 BC and BE interfacial diodes were analyzed in forward and reverse bias. A high dependence of the reverse current on the applied voltage was observed, thus obtaining rectifying ratios reaching up to 2 for BC and 17 for BE interfaces at ±0.5 V. While a previous analysis of the (n)-a-Si:H/graphene junction had yielded rectifying ratios up to 5 orders of magnitude [13], it is known that experimental values of the parameters and the quality of semiconductor interfaces are strongly affected by the fabrication process.

**Figure 2:**
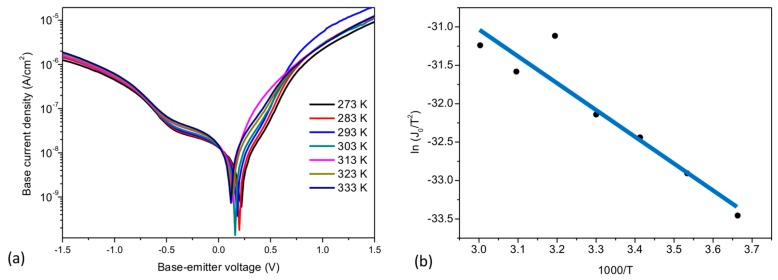
(**a**) Temperature dependent IV characteristics from 273 K to 333 K and (**b**) Richardson plot for the extraction of the base-emitter interface barrier height.

The devices were further studied by temperature dependent IV measurements to determine the Schottky barrier heights, $q\Phi_{b}$ formed at both interfaces. It was observed that the current between base and collector presented a negligible temperature dependence (not shown). This result, along with the large n value, suggests that the BC junction does not completely work as a diode, and additional transport mechanisms such as thermionic-field emission or quantum mechanical tunneling might be involved. The BE output characteristics in forward and reverse bias measured in the range from 273 K to 333 K are shown in Figure 2a. This rather narrow temperature range is restricted by the aluminum-induced crystallization of amorphous silicon at elevated temperatures [22]. At low forward bias, a temperature dependence of the current is observed. The temperature dependence of the saturation current at zero bias can be approximated as

(2) $$J_{0}\propto T^{2}exp\left(\frac{-q\Phi_{b}}{kT}\right)$$

The values of $J_{0}$ were directly extracted by extrapolating the linear part of the output characteristics to the interception voltage of 0 V. Based on Equation (2), the base-emitter Schottky barrier, $q\Phi_{b}$~0.3 eV, was determined from the slope of a $ln\left(J_{0}/T^{2}\right)$ vs 1000/T plot (Figure 2b).

**Figure 3:**
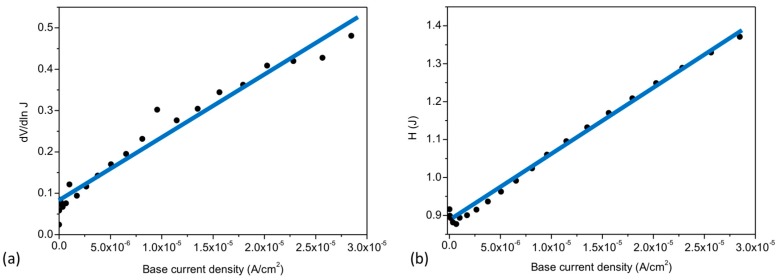
(**a**) dV/dln J versus J and (**b**) H(J) versus J plot of the forward-bias output characteristics of the BE interface.

To corroborate the obtained results, the Cheung and Cheung method [23] was used to extract the diode parameters from the experimental data in forward bias by rearranging the terms in (1), to obtain a

(3) $$\mathrm{dV}/d\ln J\;\mathrm{vs}\;J\;\mathrm{plot}\frac{dV}{d\ln\left(J\right)}=R_{s}J+nkT/q$$

and a H(J) vs J plot, where

(4) $$H\left(J\right)\equiv V-\left(\frac{nkT}{q}\right)\ln\left(\frac{J}{A^{*}T^{2}}\right)=R_{s}A_{d}J+n\Phi_{b}$$

and $A^{*}$ the Richardson constant. From the intercept and slope of the dV/dln J vs J plot (Figure 3a) the ideality factor and $R_{s}$ can be determined respectively, whereas an approximation of the Schottky barrier can be extracted from the intercept of the H(J) vs. J plot (Figure 3b). From the plots of the Cheung and Cheung method, we obtained an ideality factor of n~3.2 and a series resistance of $R_{s}$~17 kΩ. Both the ideality factor and series resistance are similar to the initial approximations. Using n and $A^{*}$ obtained from the intercept of the Richardson plot (Figure 2b) a $q\Phi_{b}$~0.27 eV for the BE interface was calculated which correlates as well with initial approximations.

Although in principle both BC and BE diodes are based on (n)-a-Si:H/graphene interfaces, the results of their electrical characterization diverge largely, which could be related to inadvertent interfacial oxide layers, surface defects and/or contaminants [24]. It must be kept in mind that the usual study case of graphene/c-Si diodes in literature is based on the transfer of graphene (lower surface) onto c-Si surfaces. However, due to the nature of CVD graphene grown on Cu foils, the lower surface of graphene in contact with the Cu foil and the upper surface might not form comparable interfaces. Lupina et al. [25] reported the presence of residual Cu atoms after the wet transfer of graphene. Ming Hong et al. [26] analyzed thin film transistors (TFT) of (n)-a-Si:H using Cu contacts and suggested that variations on the device behavior such as threshold voltage could occur due to Cu contamination in the TFT channel. Along with this, Alle et al. [27] studied the instability of a-Si:H TFTs, where water molecules have been proposed as the attacking species breaking the passivated Si bonds with H and resulting in additional interfacial traps. Thus, albeit the passivation of dangling bonds in a-Si by H, the contact of the (n)-a-Si:H collector layer with humidity cannot be excluded from the graphene transfer process which could introduce interfacial states that alter the expected barrier [28]. Therefore, surface states and deep levels in the (n)-a-Si:H induced by humidity and/or Cu residues due to graphene transfer can result in energy levels within the band gap.

In summary, the Schottky barrier of the BC diode could be largely affected by the presence of residual elements and/or interfacial states induced during the transfer of graphene onto the a-Si:H layer. At the BE interface, the (n)-a-Si:H layer was deposited on the upper surface of graphene as in previous experiments. Nevertheless, limited by the aluminum- and temperature-induced crystallization of the a-Si:H layers at temperatures larger than 150 °C [22,29,30], no annealing step was applied for the removal of possible polymer residues from the sacrificial layer [31]. This could eventually degrade the quality of the interface. Indeed, in accordance with other reports of graphene, the poor diode behavior of both junctions indicates the presence of additional transport mechanisms and/or unintentional interfacial layers [32,33].

### 3.2. Three-Terminal Characterization

**Figure 4:**
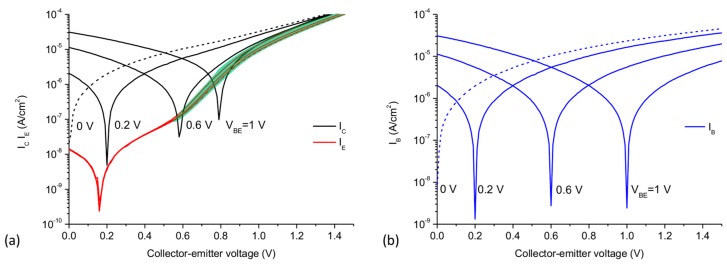
(**a**) Collector, emitter and (**b**) base currents versus $V_{CE}$. The current dips at $I_{B}$ corresponds to a leveling of the collector and base Fermi levels ($V_{BC}$ = 0 V).

Following the individual characterization of the BC and BE diodes, the test device was also analyzed in a three-terminal configuration. All currents were measured as the collector-emitter voltage $V_{CE}$ was varied at given base-emitter voltages $V_{BE}$. In Figure 4a, the measured data are presented in a semi-logarithmic plot of $I_{C}$ and $I_{E}$ versus the collector-emitter voltage. Likewise, the graphene electrode current $I_{B}$ vs $V_{CE}$ is shown in Figure 4b.

**Figure 5:**
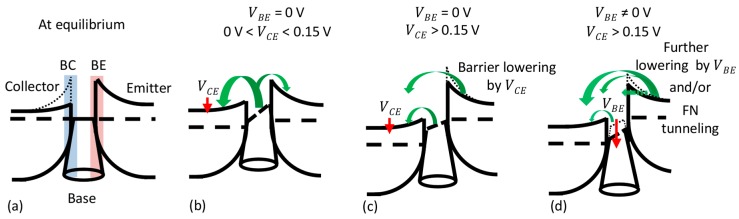
Simplified band diagrams of the graphene-base heterojunction transistor during (**a**) equilibrium (BC ideal barrier shown as a dashed line). Band diagram at $V_{BE}$ = 0 V for (**b**) 0 V < $V_{CE}$ < 0.15 V and (**c**) $V_{CE}$ > 0.15 V, and at $V_{BE}$ ≠ 0 V for (**d**) $V_{CE}\;$> 0.15 V. The green arrows indicate the flow of electrons.

First, the behavior of the device will be discussed at $V_{BE}$ = 0 V in terms of the currents ($I_{C}$, $I_{E}$ and $I_{B}$) in the voltage range 0 V < $V_{CE}$ < 0.15 V. As presented by the black dashed line in Figure 4a, $I_{C}$ increases into positive values as $V_{CE}$ increases, while $I_{B}$ increases into negative values (blue dashed line, Figure 4b). In turn, $I_{E}$ (Figure 4a red line) shows positive values of current decreasing towards $V_{CE}$ = 0.15 V and reaching a minimum at this $V_{CE}$ voltage. Thus, the electrical behavior of the device in the range 0 V < $V_{CE}$ < 0.15 V is dominated by a current flow from the base to the collector ($I_{B}$~$I_{C}$) with a small contribution of the base-emitter current as illustrated by the large and small green arrows in Figure 5b, respectively. The behavior of $I_{E}$ in this voltage range could be defined as an offset of ~0.15 V in the graphene Fermi level with respect to the emitter. Such a shift could correspond to the BE built-in potential and/or the screening effect of graphene.

As $V_{CE}$ increases above 0.15 V, the offset of the emitter to base is compensated by the applied voltage and $I_{E}$ increases exponentially with different slopes, i.e., $I_{E}$ becomes significant. This can be explained by the observed partial ohmic behavior of the BC interfacial diode, which acts as a resistor in series with the BE diode. Thus, $V_{CE}$ effectively lowers the BE Schottky-barrier (Figure 5c) and the exponential growth of $I_{E}$ can be directly attributed to a modulation of the Schottky barrier at the BE interface. Graphene monolayers have demonstrated outstanding screening capacities [34,35]. Therefore, the applied $V_{CE}$ does not fully drop along the low conductive (n)-a-Si:H layer and at the BC interface due to the low Schottky barrier. Along with this, the partial coverage of the graphene layer could act as areas of direct a-Si:H/a-Si:H contact, thus promoting the undesired control of the emitter current by the collector voltage.

In an ideal GBHT device, both BE and BC junctions exhibit significant thermionic barrier heights. The barrier heights, and thus the collector-emitter current, are controlled by the base voltage applied to the graphene layer. Since $V_{BE}$ = 0 V, the electrical performance of our device demonstrates that neither low $I_{C}$ (off) state nor saturation could be achieved, meaning that the device does not work as a conventional bipolar transistor at this point. To exemplify this case, a simplified band diagram of the GBHT in equilibrium (Figure 5a) illustrates the reduced barrier of the BC interface (the ideal barrier shown as a dashed line).

The electrical behavior of the device will now be discussed at $V_{BE}$ ≠ 0 V. For 0.2 V < $V_{BE}$ < 1 V. The current flow is still dominated by a base-collector ($I_{B}$~$I_{C}$) current, i.e., $I_{E}$ has a negligible dependency to the base voltage even during forward bias conditions of the BE diode. This behavior is caused again by the ohmic behavior of the BC junction, the low bulk resistance of the collector layer and/or the diode efficiencies of the junctions. At all applied $V_{BE}$, $I_{C}$ and $I_{B}$ reach a minimum (current dip) before changing to positive and negative values respectively. The current dip position of $I_{B}$ corresponds to the applied $V_{BE}$ and can be understood by leveling the Fermi levels, i.e., collector-base voltage is 0 V. The visible “V” shape characteristics of $I_{B}$ is caused by a change of the current flow due to the large leakage currents of the BC junction (an ideal diode would allow only a low reverse current). The current dip of $I_{C}$ demonstrates a different behavior. As $V_{CE}$ raises above 0.5 V, the exponential increase of $I_{E}$ prevents the $I_{C}$ current dip from coinciding with the applied $V_{BE}$, i.e., at $V_{BC}$ = 0 V.

**Figure 6:**
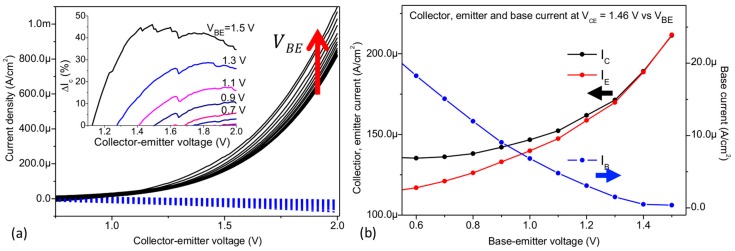
(**a**) Base (blue dashed lines) and collector (black solid lines) current versus $V_{CE}$ at constant values of $V_{BE}$ ranging from 0 to 1.5 V in 100 mV steps. Inset: Percentage increase of $I_{C}$ in function of $V_{BE}$ in respect to $I_{C}$ at $V_{BE}=\;$0 V. (**b**) Variation of the collector, emitter and base currents extracted at $V_{CE}=$ 1.46 V in function of the graphene base voltage.

At $V_{CE}$ values above 0.5 V, an increment of the emitter current as function of $V_{BE}$ can be observed (green shade in Figure 4a). This area indicates the collector-emitter current modulation by the base voltage, and thus demonstrates a first step towards an operational GBHT. The small modulation of the collector-emitter current is illustrated in Figure 6a, where $I_{C}$ and $I_{B}$ are linearly plotted while varying the collector-emitter voltage $V_{CE}$ at different constant base-emitter voltages $V_{BE}$ ranging from 0 V to 1.5 V. At $V_{BE}$ = 0 V, the collector current $I_{C}$ is still highly affected by $V_{CE}$. However, the variation of the collector current caused by $V_{BE}$ can be highlighted. The increase of the collector current ($\Delta I_{c}$), defined as the change of $I_{C}$ at $V_{BE}$ = 0 V vs $V_{BE}$ ≠ 0 V, can reach up to 40% as seen in the inset of Figure 6a.

In Figure 6b, all currents were extracted at $V_{CE}$ = 1.46 V (the largest percent of variation) and plotted versus the base-emitter voltage. $I_{B}$ steadily decreases as $V_{BE}$ increases, while the collector and emitter current increases at the same time, i.e., the operation is mainly dominated by a vertical collector-emitter transport through the base. The observed modulation of the collector-emitter current by the graphene base voltage, in a purely thermionic transport, could be understood as a further reduction of the Schottky barrier at the BE interface. However, due to the relatively large electric fields at which the current gain takes place, additional transport mechanisms, such as Fowler-Nordheim (FN) tunneling [36], could be involved. Indeed, Mouafo et al. [37] investigated the temperature dependent IV characteristics of Ti/MoSe_2_ junctions and found a transition of the main transport mechanism from thermionic emission to FN tunneling around 1 V bias. Similarly, FN tunneling could occur in our vertical a-Si:H/graphene structure. However, it must be noted that differently from the gated Ti/MoSe_2_ of ref. [37], where the width of the space charge region is limited by the 2D crystal and controlled by the gate, the space charge region of the a-Si:H/graphene interface is much wider (~44 nm, as measured by capacitance-voltage characterization by Strobel et al. [13]). Therefore, direct or FN tunneling mechanisms seem unlikely. Although a more detailed and extended analysis is required in future work to fully understand the involved current transport mechanisms, the current gain can be seen as a further reduction of the barrier which promotes purely thermionic and possibly FN tunneling transport which add an extra electron flow on $I_{E}$ as depicted in Figure 5d. In addition, since the applied voltages are close to the band gap of a-Si:H ($E_{g}/e$~$V_{CE}$), the transport through the valence band i.e., band-to-band tunneling or impact ionization could be expected [4,38]. The largest extracted transconductance value $g_{m}=dI_{C}/dV_{BE}$ was ~230 μS, demonstrating a moderate modulation of the collector-emitter current by the ultra-thin graphene base voltage.

## 4. Conclusions

In conclusion, we presented the electrical characterization of the interfacial diodes in (n)-a-Si:H/graphene/(n^+^)-a-Si:H heterostructures in the vertical GBHT configuration. We found that the collector-base diode is characterized by a small Schottky barrier and low rectifying ratios, which could be caused by atomic residues and/or humidity-induced interfacial states. The base-emitter diode is characterized by a rectifying ratio of more than one order of magnitude and a small Schottky barrier $q\Phi_{b}$~0.3 eV. The poor diode-like characteristics of the BC junction yield large leakage currents and thus an unconventional transistor behavior was achieved by the three-terminal operation. Along with these characteristics, the collector voltage does not completely drop at the interface due to the low BC Schottky barrier, effectively lowering the BE barrier and generating an undesired emitter current even in the absence of a base-emitter voltage. Nevertheless, it was experimentally demonstrated that the bias voltage applied at the embedded ultra-thin graphene base modulates the vertical current up to 40%, due to a modest control of the BE barrier.

Enhancing the current control in vertical transistor structures could be achieved by further optimizing the diode behavior of the interfaces, specifically the lack of rectification at the base-collector junction. A possibility to increase the barrier at the BC interface is by reducing the doping of the collector layer. Along with this, the quality of the BC interface could be improved by avoiding atomic Cu residues and humidity-related issues. This could be achieved, e.g., by the *dry-transfer* of graphene layers grown on germanium substrates [39]. Another route to improve the transistor behavior of the device, specifically the off-state, could be based on the insulating states provided by bilayer graphene. These states are caused in bilayer graphene by an electric field applied normal to the plane [40]. Since such conditions are natural in the vertical architecture of the GBHT, it presents a feasible and promising object of future investigations. Other 2D materials can be considered without losing the advantage for flexible electronics. For example, transition metal dichalcogenides, such as MoS_2_, MoSe_2_ or WSe_2_, have been extensively used in gate-tunable heterojunctions or in field effect transistor reaching high on/off ratios of up to 10^7^ [37,41]. However, this approach might present other challenges such as the drastic reduction of mobility [42], compared to that of graphene, which could have a great impact on the maximal transition frequency of the vertical transistor.
